# Nutrient foramen location on the laminae provides a landmark for pedicle screw entry: a cadaveric study

**DOI:** 10.1186/s12891-018-2218-0

**Published:** 2018-08-16

**Authors:** Masahito Oshina, Yasushi Oshima, Yoshitaka Matsubayashi, Yuki Taniguchi, Hirotaka Chikuda, Kiehyun Daniel Riew, Sakae Tanaka

**Affiliations:** 1 Department of Orthopaedic Surgery, The University of Tokyo Hospital, 7-3-1, Hongo, Bunkyo-Ku, Tokyo, 113-8655 Japan; 2 Department of Orthopedic Surgery, Columbia University, New York, NY USA

**Keywords:** Nutrient foramen, Laminae, Pedicle screw, Entry point, Cervical spine, Thoracic spine

## Abstract

**Background:**

Nutrient foramina are often encountered around the entry point of pedicle screws. Further, while probing the pedicle for pedicle screw insertion around the nutrient foramen, bleeding from the probe insertion hole is often observed. The purpose of this study was to investigate the frequency of occurrence of nutrient foramina, the association between the nutrient foramen and pedicle, and the safety and accuracy of cervical and thoracic pedicle screw placement using the nutrient foramen as the entry point.

**Methods:**

We identified the location of the nutrient foramina for the dorsal branches of the segmental artery and their anatomical association to the pedicles and bony landmarks in the vertebrae for C3–T12 in seven cadavers. We also determined the frequency with which the nutrient foramina were present in 119 cadaveric vertebrae. We identified the pedicle location, base of the superior articular facet, and lateral border of laminae with respect to the nutrient foramen.

**Results:**

The overall presence of the nutrient foramina was 63% (150/238) in the specimens, with 60% (42/70) and 64% (108/168) identifiable in the cervical and thoracic vertebrae, respectively. In the cervical vertebrae, the nutrient foramen was located on the outer wall of the pedicle and was positioned between the cephalad and caudal walls. In the thoracic spine, 98% (106/108) nutrient foramina were located inside the pedicle walls.

**Conclusions:**

Our study findings confirm that the location of the nutrient foramen can be used for identifying the entry point for pedicle screws. In the cervical vertebrae, the nutrient foramina are located lateral to pedicle but within the cranial and caudal margins. In the thoracic vertebrae, the nutrient foramina are located in the medial and caudal regions of the pedicle. Thus, to decrease the risk of overshoot, the entry point for thoracic pedicle screws should be positioned a few millimeters cephalad and lateral to the nutrient foramen.

## Background

Pedicle screws have been used since Boucher [1] reported this technique for the lumbar spine in 1959. Several entry points for pedicle screws have been described, but the risk of neural, vascular, or visceral injury remains [2–8]. The safety of screw placement can be improved if intraoperative fluoroscopy and computed tomography (CT) and image-assisted navigation are employed [9–12]. However, these techniques do not completely eliminate the risk of injury. Therefore, obtaining information that can help in improving the accuracy of pedicle screw placement is desirable.

Nutrient foramina are often encountered around the entry point of pedicle screws. Further, while probing the pedicle for pedicle screw insertion around the nutrient foramen, bleeding from the probe insertion hole is often observed. These nutrient foramina are considered to be the entry points for the dorsal branch of segmental arteries, and they have a predictable location on the laminae [13]. A previous study described the details of intravertebral vasculature following radiopaque dye injection [14], but the positional association of the vasculature to the pedicles has not yet been reported. This study was performed to determine the frequency of occurrence of nutrient foramina, the association between the nutrient foramen location and pedicle and other bony landmarks, and the safety and accuracy of cervical and thoracic pedicle screw placement using nutrient foramina as the entry point.

## Methods

We used seven cadavers (four male and three female) for this study. The mean age of the cadavers at the time of death was 87.9 years. The cadavers were provided by the Department of Anatomy (The University of Tokyo, Japan). In total, 238 pedicles and nutrient foramina of the dorsal branches of segmental arteries between the C3 and T12 were evaluated manually. The cadavers were placed in the prone position with the neck in the neutral position. Soft tissue was removed to expose the laminae, facet joints, and transverse processes.

**Fig. 1:**
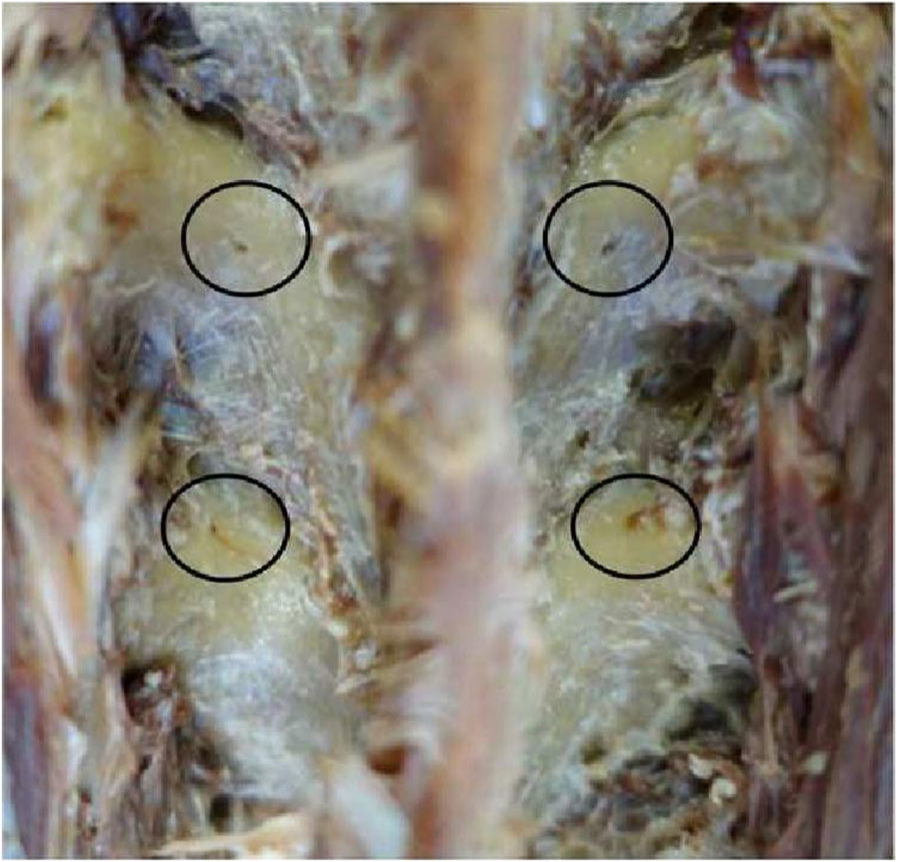
Nutrient foramen on laminae

We manually examined each cadaver for the presence of nutrient foramina for the dorsal branches of the segmental artery around the superior articular facet and transverse processes. The presence of a nutrient foramen was confirmed by the identification of a circular depression with a lack of cortical continuity (Fig. 1).

**Fig. 2:**
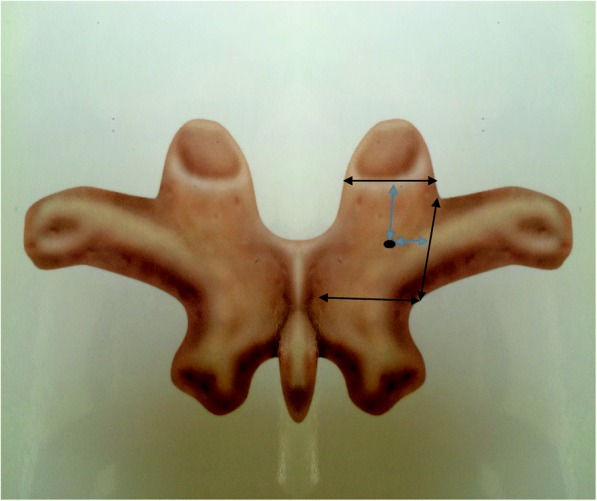
Nutrient foramina in the studied area and distance from the nutrient foramen to the bottom of superior articular facet and the lateral border of laminae

If a nutrient foramen was present, the distance from the lateral border of the lamina and the bottom of the superior articular facet was measured (Fig. 2). The nutrient foramina were found to be usually located just caudal to the superior articular facet and medial to the base of the transverse process on the border of the laminae and cranial to the inferior margin of the transverse process. If two nutrient foramina were present in this area, the larger one was evaluated. Nutrient foramina in other areas were excluded (Fig. 2).

A Kirschner wire measuring 1.2 mm in diameter (TACT MEDICAL INC., Tokyo, Japan) was orthogonally inserted through the nutrient foramen toward the vertebral body without changing direction in the sagittal and axial planes. The Kirschner wire was used instead of a pedicle screw so that the nutrient foramen and the canal remained intact. After the nutrient foramen was marked, the lamina was cut using a chisel. To inspect the inside of intact pedicles, some nutrient foramina were evaluated by cutting the pedicle with a chisel at the foramen without Kirschner wire insertion. The association between the nutrient foramen and pedicle positions was examined on the C3–T12 laminae.

All measurements were made using an electronic digital caliper (precision 0.01 mm; PLATA, Osaka, Japan). We manually measured four anatomic parameters related to the mouth of the dorsal branch of the segmental artery on the C3–T12 laminae. The same caliper was used to measure the distance between the nutrient foramina and various structures. The following parameters were assessed:

1. Percentage of occurrence of nutrient foramina. The area of evaluation was determined using definite bony landmarks.
2. Caudally directed distance from the bottom of the superior articular facet.
3. Medially directed distance from the lateral border of the lamina.
4. Assessment of whether the orthogonal line of lamina at a nutrient foramen was located inside or outside the pedicle, with the location determined when it was located inside the pedicle wall.

When there were two or more exposed foramina, the distance between the nutrient foramen and the bony landmark was compared between the left and right sides using Student’s *t*-test. Differences with *P* values of < 0.05 were considered statistically significant.

## Results

Nutrient foramina were present within the evaluation area in 60% (42/70) of cervical vertebrae and 64% (108/168) of thoracic vertebrae. Overall, nutrient foramina were present in 63% of vertebrae (150/238; Fig. 1 and Tables 1 and 2). There were no significant differences bilaterally in the distance between the nutrient foramen and bony landmarks.

**Table 1:** Number of exposed nutrient foramina and mean distance from foramina to the bony landmark

	Exposure (/14)	Lt (/7)	Rt (/7)	Lt. superior articular facet (mm)	Lt. border of the laminae (mm)	Rt. superior articular facet (mm)	Rt. border of the laminae (mm)
C3	9	4	5	2.51 ± 1.18	4.70 ± 1.33	2.96 ± 0.76	4.12 ± 1.92
C4	6	2	4	2.84 ± 5.57	3.74 ± 0.07	2.57 ± 0.38	3.82 ± 0.48
C5	9	6	3	3.08 ± 1.27	5.28 ± 1.73	3.79 ± 1.52	6.27 ± 1.35
C6	10	4	6	2.76 ± 0.37	4.82 ± 1.28	3.04 ± 0.61	4.86 ± 1.00
C7	8	4	4	2.93 ± 0.45	6.81 ± 0.86	3.63 ± 0.43	6.67 ± 0.67

**Table 2:** Number of exposed foramina and mean distance from the foramina to the bony landmark

	Exposure (/14)	Lt (/7)	Rt (/7)	Lt. superior articular facet (mm)	Lt. border of the laminae (mm)	Rt. superior articular facet (mm)	Rt. border of the laminae (mm)
T1	12	6	6	4.09 ± 0.52	3.64 ± 0.63	4.09 ± 0.71	3.75 ± 0.68
T2	11	5	6	4.44 ± 1.55	3.99 ± 0.86	4.30 ± 1.24	4.24 ± 1.02
T3	8	4	4	4.71 ± 1.01	3.99 ± 0.46	4.69 ± 0.89	4.27 ± 0.24
T4	8	4	4	4.07 ± 1.21	4.07 ± 0.53	3.62 ± 1.48	3.91 ± 0.42
T5	4	3	1	3.67 ± 0.26	3.61 ± 0.72	5.74	4.40
T6	8	5	3	4.44 ± 1.23	4.02 ± 0.36	4.44 ± 1.21	3.80 ± 0.60
T7	7	4	3	3.54 ± 0.45	4.23 ± 0.17	3.49 ± 0.28	3.90 ± 0.53
T8	10	5	5	3.55 ± 0.54	4.49 ± 1.25	3.38 ± 0.71	4.18 ± 0.89
T9	8	4	4	3.80 ± 0.90	4.64 ± 1.80	4.11 ± 0.79	4.08 ± 0.49
T10	12	5	7	4.20 ± 0.81	4.08 ± 0.43	3.64 ± 0.95	4.27 ± 0.35
T11	8	4	4	4.03 ± 0.23	3.28 ± 1.19	3.56 ± 0.92	3.67 ± 1.13
T12	12	6	6	4.65 ± 1.37	2.67 ± 0.58	4.28 ± 1.54	2.84 ± 0.53

### Association between the superior articular facet and the nutrient foramen

When making calculations, we excluded the nutrient foramina on the superior articular facet and those located caudally to the transverse process (Tables 1 and 2). The distance from the base of the superior articular facet to the nutrient foramen had a range of 1.28–4.98 mm in the cervical vertebrae and 2.22–6.50 mm in the thoracic vertebrae. The distance from the base of the superior articular facet to the nutrient foramen was similar between the cervical and thoracic vertebrae.

### Association between the base of the transverse process and the nutrient foramen

The distance from the lateral laminar border and base of the transverse process to the nutrient foramen was 3.14–7.25 mm in the cervical vertebrae and 2.01–7.32 mm in the thoracic vertebrae. The distance tended to be similar between the cervical and thoracic vertebrae (Tables 1 and 2).

In the cervical vertebrae, the nutrient foramen was usually located inside the vertebral laminar notch. In the thoracic vertebrae, no nutrient foramen was located medial to the inflection point, where the lamina meets the transverse process. At the T11 and T12 levels, nutrient foramina were located just inside the accessory process and tended to be close to the lateral laminar border. It may be considered that the nutrient foramen position on the laminae moves caudally at this level, probably because laminae are narrower.

### Association between the pedicle and nutrient foramen

In the cervical spine, almost all Kirschner wires inserted into the nutrient foramen reached the outer aspect of the pedicle and were located immediately above the course of the vertebral artery. However, at the C7 level, the wires reached beyond the outer aspect of the vertebral artery. In the cervical spine, two nutrient foramina in C3 vertebra deviated vertically from the pedicle axis to the caudal direction, whereas the others were located in the cephalad and caudal margins.

**Fig. 3:**
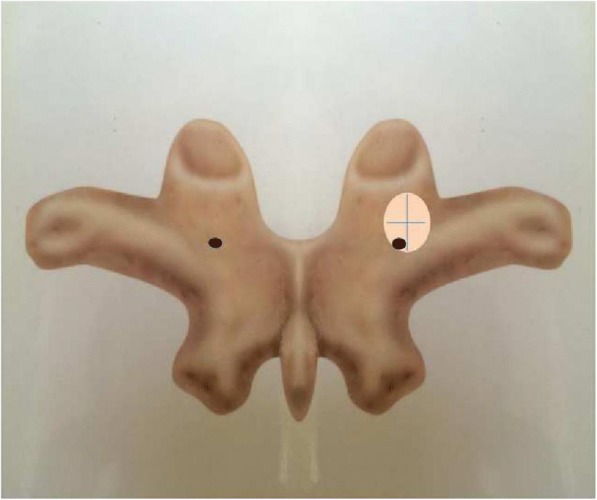
In most cases, nutrient foramina existed in the medial caudal side of pedicle

**Fig. 4:**
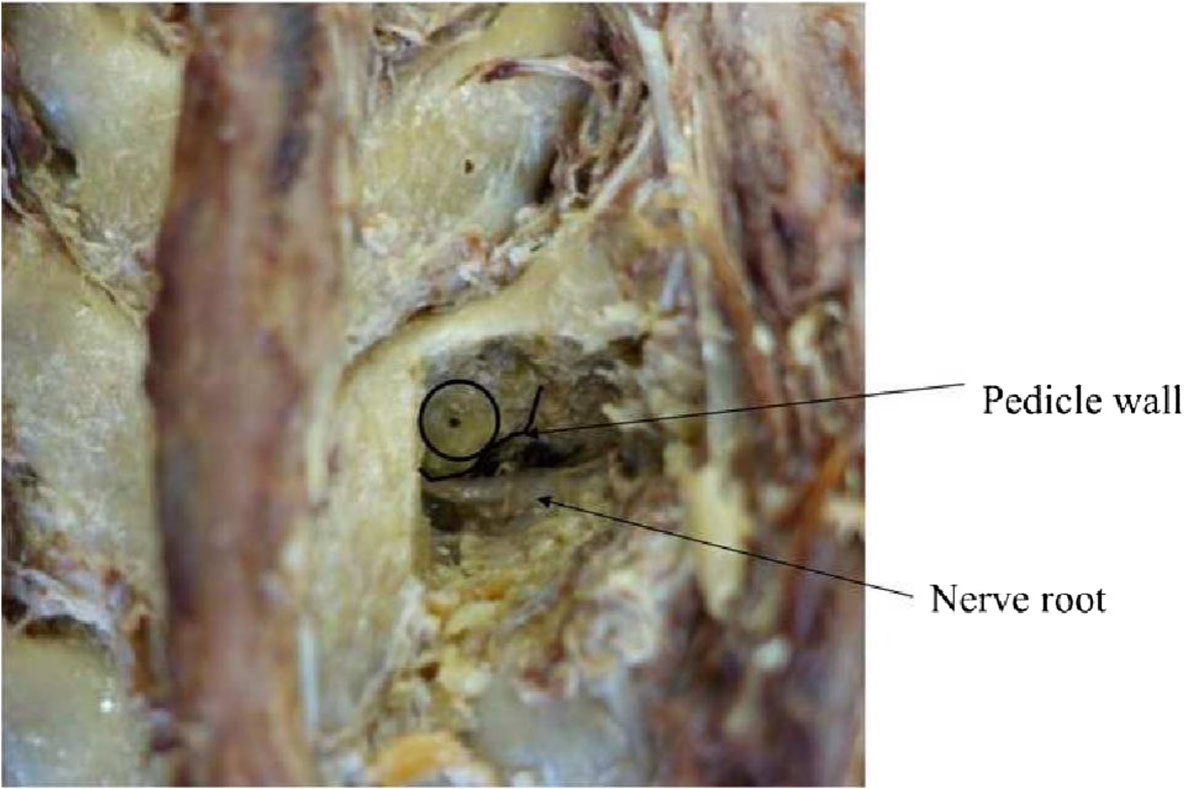
Nutrient foramen penetrating deep into the pedicle

In the thoracic spine, two nutrient foramina in T2 vertebra deviated from the pedicle axis to the caudal direction. However, the deviated nutrient foramina were located within the pedicle width, and no nutrient foramen was observed to perforate the medial pedicle wall. The remaining nutrient foramina were all located on the medial and caudal sides of the pedicle (Fig. 3). Some nutrient foramina were observed in the inner aspect of pedicles after laminae were cut (Fig. 4).

In total, 98% (106/108) of thoracic nutrient foramina were located within the margins of the pedicle walls. In addition, some nutrient foramina located on the superior articular facet were also within the margins of the pedicle wall. However, they were excluded because the location was out of the investigational range. The nutrient foramina situated below the transverse process were outside the margins of the pedicle walls.

## Discussion

The segmental artery gives rise to smaller branches that supply the vertebral body in its proximal portion. Three types of branches exist: ventral, dorsal, and spinal. The course of the dorsal branch is sub-laminar before it perforates the muscles [14, 15]. The nutrient foramina for the dorsal branches of the segmental artery were evaluated in this study. We found that the foramina were located close to the entry point for pedicle screws in almost all specimens, and we investigated the frequency of its presence and its positional association to the pedicle. When the nutrient foramen was cut, we were able to observe the path of the vessel, which went through the cortical bone into the pedicle, continuously on the outer aspect of the laminae [14] (Fig. 4). Although obvious pathways are depicted in Fig. 4, it is unclear whether all nutrient foramina pass through the pedicle. This is because the smaller pathways could have been destroyed and compressed when we cut the bone using the chisel. In addition, when nutrient foramina and vessel pathways are small, it may be difficult to identify them. In this study, we visually identified the presence and location of nutrient foramina on the laminae by careful examination. Another method for identifying the nutrient foramina is CT [16]. Nutrient foramina can often be confirmed using three-dimensional CT (3DCT), but the confirmation depends on the size of the nutrient foramen and whether it was captured on a slice. Therefore, this technique cannot assess the presence of a nutrient foramen with sufficient detail and accuracy, without very fine slice CT scans. Thus, the confirmation of screw insertion into a nutrient foramen via CT or navigation system can only be performed when CT is able to accurately visualize the foramina. However, with very fine slice CT scans, one can validate the technique that we used in this study. We are currently in the process of conducting such a study.

### In the cervical spine

With regard to the rate of nutrient foramen occurrence and its association to bony landmarks, if facet joint hypertrophy is relatively severe, the identification of the nutrient foramen becomes difficult because the foramen gets closer to the enlarged facet and is covered by an overlapping osteophyte. This issue is particularly the case in the cervical spine. In such cases, the nutrient foramen can still be used as a landmark for the entry point for pedicle screws, but the direction of insertion should be medially directed. For example, when we determine the trajectory of the pedicle screw, this entry point should be selected considering the medially inclined pedicle axis, as reported previously [2, 17]; this is because the nutrient foramen is located immediately above the vertebral artery. In the sagittal plane, the trajectory should be orthogonal to the dorsal spine curvature; thus, the C3 insertion trajectory is expected to deviate caudally. Rao et al. [18] reported that sagittal pedicle angulation was directed cranially at C3 (13.9°) and C4 (7.3°). As noted in previous studies [18, 19], accuracy may be enhanced if the cephalad direction is used at the C3 and C4 vertebrae.

### In the thoracic spine

With regard to the rate of nutrient foramen exposure, in contrast to the cervical vertebra, the thoracic vertebrae were less degenerative, and the thoracic nutrient foramina were consistently identifiable. Nutrient foramina were almost at the inflection point between the lamina and transverse process. Nutrient foramina tended to be present in a more cranial position at T7 and T8, suggesting that the pedicles are also positioned more cranially, which is similar to that previously reported [18–20]. Nutrient foramina located within the margins of the pedicle (106/108) were located in the medial and caudal portion of the pedicle. Therefore, thoracic pedicle screw entry points should be positioned a few millimeters cephalad and lateral to the nutrient foramen. Mid-thoracic pedicles are usually vertical and do not require an extreme medial angulation.

Our study has several limitations. The number of cadavers included was small, making it difficult to compare the location of the nutrient foramen at each level by statistical analysis. Increasing the number of cadavers should increase the accuracy of the assessments. Similar to our study, Yang, et al. [21] have reported that nutrient foramina were present in 63% from T4 to T8 vertebrae. The difference of presentation among other ethnicities and Asian is remains unclear because their study did not take into account the race of the cadavers. However, as a pilot study for identifying 42 cervical and 108 thoracic nutrient foramina, we believe that we have adequate data to demonstrate the association between the nutrient foramina and pedicles in the cervical and thoracic vertebrae. Another limitation is that one cannot rely on the nutrient foramina as a guide for placing pedicle screws in isolation. To date, no foolproof technique has been identified for placing cervical and thoracic pedicle screws. We believe that the greatest utility of the nutrient foramina is that when present and when identified on a 3DCT image, one has a perfect intraoperative landmark to use as a guide for inserting the pedicle screws. One can use a preoperative navigation software to determine the ideal starting point, in addition to using the nutrient foramen as an intra-operatively identifiable landmark.

## Conclusion

Our study results indicate that the nutrient foramen is identifiable in the majority of cervical and thoracic vertebrae and that it is in close proximity or within the margins of the pedicle walls. The location of the nutrient foramen was consistent, especially in the thoracic spine. The cervical nutrient foramina were located lateral to pedicle, but within the cranial and caudal margins. The thoracic nutrient foramen is most commonly located inside of the pedicle wall, and it is positioned in the medial and caudal aspect of the pedicle. Thus, to provide a smaller overshoot risk, although prior confirmation by CT is needed, the thoracic pedicle screw entry point should be positioned a few millimeters cephalad and lateral to the nutrient foramen. Most importantly, we believe that if the nutrient foramen can be identified on a 3DCT image, it can be used, along with a navigation software or freehand technique, to pre-operatively plan the starting point and trajectory of a pedicle screw using the nutrient foramen as a reference point.

## Abbreviations

3DCT: Three-dimensional computed tomography
CT: Computed tomography
